# A nonlinear relationship between systemic inflammation response index and short-term mortality in patients with acute myocardial infarction: a retrospective study from MIMIC-IV

**DOI:** 10.3389/fcvm.2023.1208171

**Published:** 2023-07-24

**Authors:** Yufei Wang, Hua Chen

**Affiliations:** ^1^ Graduate School, Inner Mongolia Medical University, Hohhot City, China; ^2^ Department of Cardiology, Inner Mongolia Autonomous Region People's Hospital, Hohhot City, China

**Keywords:** systemic inflammation response index, acute myocardial infarction, predictor, allcause mortality, medical information mart for intensive care-IV

## Abstract

**Background:**

This investigation aimed to evaluate the efficacy of the Systemic Inflammatory Response Index (SIRI) in prognosticating short-term all-cause mortality among patients diagnosed with acute myocardial infarction (AMI) in the intensive care unit (ICU).

**Methods and Results:**

Clinical data were obtained from the Medical Information Mart for Intensive Care-IV (MIMIC-IV) database. A total of 4,291 patients were included in the cohort. Results from multivariate regression analyses showed that the quartile of the natural logarithm of SIRI (ln-SIRI) was independently associated with mortality. Compared to patients in the first quartile (Q1), patients in the second quartile (Q2) and fourth quartile (Q4) were significantly associated with an increased risk of 30-day (HR = 2.031, 95% CI: 1.604–2.571, *p* < 0.001 and HR = 1.703, 95% CI: 1.32–2.195, *p* < 0.001) and 90-day all-cause mortality (HR = 2.063, 95% CI: 1.68–2.532, *p* < 0.001 and HR = 1.788, 95% CI: 1.435–2.227, *p* < 0.001), which is consistent with the results of the Kaplan-Meier analysis and the results of multivariate regression analyses by classifying into 12 groups based on dodeciles of SIRI. Curve fitting showed a curvilinear relationship and further threshold saturation effects showed that, for 90-day mortality, each unit increased in ln-SIRI, when the ln-SIRI level is less than 2.9, the patient's mortality increases by 23.2% (OR: 1.232; 95% CI: 1.111–1.367; *p* < 0.001); when the ln-SIRI is greater than 2.9 and less than 4.6, the patient's mortality decreases by 44.4% (OR: 0.554; 95% CI: 0.392–0.789; *p* = 0.001); when ln SIR > 4.6, the patient's mortality increases by 24.7% (OR: 1.247; 95% CI: 1.108–1.404; *p* < 0.001). Moreover, the length of stay in the hospital was lower in patients in the third quartile (Q3) (coefficient: −1.999; 95% CI: −2.834 – −1.165, *p* < 0.001). The length of stay in the ICU was higher in patients in Q2 and Q4 (coefficient: 0.685;95% CI: 0.243–1.128; *p* = 0.0024 and coefficient: 0.989;95% CI: 0.528–1.451; *p* < 0.001). Furthermore, SIRI may outperform NLR in predicting short-term mortality.

**Conclusion:**

SIRI is an independent risk factor for 30- and 90-day mortality, and length of stay in ICU for critical AMI patients.

## 1. Introduction

Acute myocardial infarction (AMI) is a prevalent and severe cardiovascular ailment that afflicts individuals worldwide. In recent times, the incidence rate of this condition has surged, and it has shown a predilection for younger patients. AMI can lead to cardiac ischemia and hypoxia, which can prove to be life-threatening (1). A report has demonstrated that the in-hospital mortality rate for ST-elevation myocardial infarction (STEMI) is approximately 9.2% (2). The risk of mortality in patients with AMI within one-year ranges between 4% and 12% (3). The burden of AMI is escalating due to an aging society, which exerts pressure on patients, their families, and society, particularly among Intensive Care Unit (ICU) patients. Identifying prognostic factors that can predict patients with the highest risk of adverse outcomes is imperative. Novel and exceptional risk markers should be simpler and more cost-effective, provide more comprehensive information, and have a clear pathophysiological basis.

The Systemic Inflammatory Response Index (SIRI) is a composite indicator that is calculated by peripheral neutrophil count, monocyte count, and lymphocyte count. This innovative indicator boasts simple detection, strong practicability, and low cost. Previous studies have demonstrated that SIRI is an independent prognostic indicator for various malignant tumors, including cervical cancer (4). Furthermore, SIRI has been linked to the activity and progression of rheumatic diseases, such as rheumatoid arthritis (5). Additionally, SIRI has been identified as an independent predictive indicator for patients with acute ischemic stroke caused by large artery occlusion after mechanical thrombectomy (6). In the case of Intracerebral hemorrhage patients, SIRI has been shown to be an independent predictive indicator for 3-month functional outcomes and 1-month mortality, with a stronger prognostic predictive ability than NLR (7).

However, studies on SIRI and AMI are still relatively few and the results are not clear enough. In this study, we explored the relationship between SIRI and the risk of death in patients with AMI in the intensive care unit based on the Medical Information Mart for Intensive Care (MIMIC)-IV database.

## 2. Methods

### 2.1. Data sources and study population

**Figure 1:**
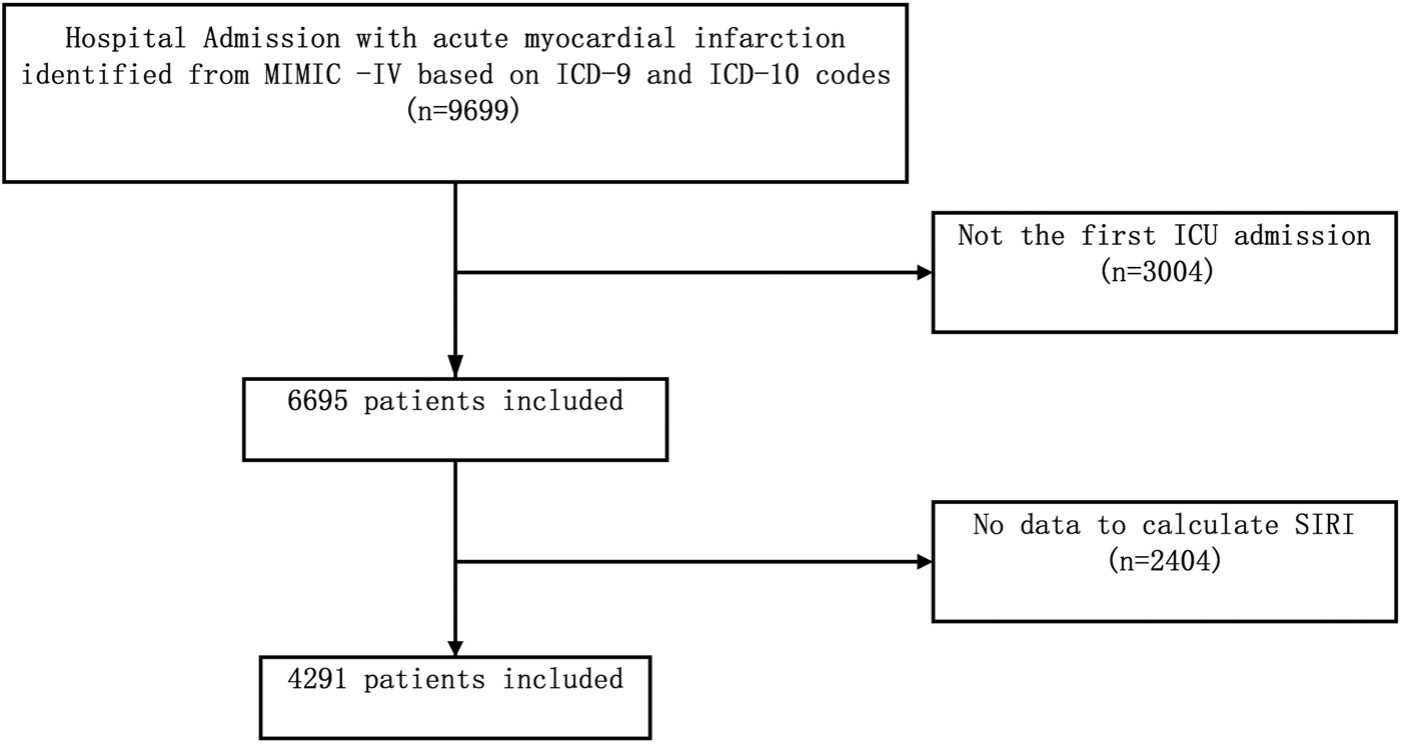
Flow chart of study population selection. ICU, intensive care unit; MIMIC- IV, Medical Information Mart for Intensive Care-IV; SIRI, systemic inflammation response index.

The retrospective cohort study was designed according to the Strengthening the Reporting of Observational Studies in Epidemiology guidelines (8). All patient information used in the study was extracted from the Medical Information Mart for Intensive Care IV (MIMIC-IV 2.2) database. This real-world public database was created using inpatient data from 2012 to 2019 from Beth Israel Deaconess Medical Center in Boston, Massachusetts, USA. It contains demographic data, data on various laboratory tests, and operations performed during hospitalization. The database also includes specific dates of death for patients from admission to one-year post-discharge, which allows investigators to study clinical indicators related to mortality. One author of this article, Yufei Wang, passed the “Protecting Human Research Participants” examination on the National Institutes of Health (NIH) website. A data usage agreement (Record ID: 53670131) was signed, granting access to the database. The clinical data used in this study did not require ethical approval and informed consent because information related to patient privacy in the database has been anonymized. Of the 257,366 people recorded in the MIMIC-IV database, 50,048 were admitted to the ICU. The search was restricted to adult AMI patients admitted to the ICU for the first time, using 410 in ICD-9 and I21 in ICD-10 codes. Patients without key variables (absolute lymphocyte count, absolute monocyte count, absolute neutrophil count) for analyses were excluded. After excluding duplicate data, a total of 9,699 patients were diagnosed with AMI. Of these, 4,291 patients met the criteria and were included in this study (Figure 1).

### 2.2. Variables

This study utilized Structured Query Language (SQL) through PostgreSQL software for data extraction. SIRI is a continuous variable calculated by multiplying the neutrophil count by the monocyte count and then dividing by the lymphocyte count. To eliminate skewness in the independent variable SIRI, we performed a natural logarithm transformation on it. Specifically, we took the natural logarithm of SIRI to obtain a new variable named ln-SIRI, which was used in place of the original independent variable for subsequent analysis.

Multiple covariates were considered in this study. Demographic variables and vital signs were extracted from the database, including age, gender, ethnicity, systolic blood pressure (SBP), diastolic blood pressure (DBP), mean blood pressure (MBP), body temperature, heart rate, respiratory rate (RR), and peripheral oxygen saturation (SpO2). Information on the patient's comorbidities was also extracted, including hypertension, diabetes, cerebrovascular disease, peripheral vascular disease, congestive heart failure, rheumatic disease, chronic obstructive pulmonary disease, kidney disease, severe liver diseases, hypercholesterolemia, ventricular tachycardia, and malignant neoplasm. Additionally, laboratory variables were collected from the database, including white blood cell (WBC) count, platelet count, hemoglobin, glucose, sodium, calcium, chloride, creatinine, and partial thromboplastin time (PTT). Data on drug use after admission to the ICU, including warfarin, aspirin, and beta-blockers, were also obtained.

To maintain statistical power and diminish bias that might occur after subjects with missing data were excluded from analyses, we used multivariate multiple imputation with chained equations to impute missing values (9). We repeated all analyses with the complete data cohort for comparison. Additional details of the statistical analyses were given in Supplementary materials.

### 2.3. Outcomes

To study the short-term mortality of patients, the primary endpoint of this study was set as 90-day all-cause mortality. Secondary outcomes included 30-day all-cause mortality, 30- and 90-day all-cause re-admission, length of stay in hospital, and length of stay in ICU.

### 2.4. Statistical methods

The baseline clinical characteristics of the patients were stratified into four groups based on the quartile of SIRI. Theņ the data were categorized into discrete and continuous variables. The continuous variables were further subdivided into two categories based on their normality distribution. Normally distributed continuous variables were presented as mean and standard deviation, and compared between groups using the Student's *t*-test. Non-normally distributed data were reported as the median and interquartile range (IQR), and compared between two groups using the Wilcoxon rank-sum test. Categorical variables were expressed as percentages and compared between two groups using the chi-square test. The Kruskal-Wallis test or one-way analysis of variance was used to evaluate the statistical significance of differences between groups stratified by quartile of ln-SIRI.

We conducted univariate and multivariate Cox regression analyses to explore the correlation between ln-SIRI and the SIRI quartile and short-term mortality and short-term readmission. Two models were developed to adjust for confounding factors. Model 1 adjusted for demographic variables, including sex, age, and ethnicity, which were collected prior to the study. Model 2 adjusted for demographic variables as well as vital signs (heart rate, systolic blood pressure, mean blood pressure, respiratory rate, and percutaneous oxygen saturation), laboratory variables (hemoglobin, calcium, sodium, and partial thromboplastin time), complications (diabetes, congestive heart failure, chronic obstructive pulmonary disease, malignant cancer, hypercholesterolemia, and hypertension), and drug use information (aspirin, warfarin, beta-blockers). The adjusted variables were consistent with Models in the multivariate Cox regression analysis. We adjusted for potential confounders based on two criteria: (1) confounders identified in previous studies, and (2) covariates resulting in a change of greater than 10% in effect values during covariate screening. The Kaplan–Meier(K–M) curve was also utilized to visualize the relationships between the SIRI quartile and short-term mortality.

To validate the stability of the results of multivariate Cox regression analyses, other analyses were utilized. The SIRI was divided into 12 groups based on dodeciles, and a Cox multivariate regression analysis was performed, with adjusted covariates consistent with Model 2 in the multivariate regression analysis. To clearly visualize the corresponding results, the results are presented using forest plots. Smooth curve fitting and cubic spline functions were used to construct a Cox proportional-hazard regression model to examine the likelihood of a non-linear association between ln-SIRI and mortality. By highlighting characteristics that may be overlooked using classification approaches, this curve fitting enables a thorough investigation of the relationship between ln-SIRI and mortality. Based on the non-linear relationship observed in the preliminary analysis, a three-piecewise linear regression model was used for finding threshold effects. This model allowed us to identify the optimal threshold values by maximizing the likelihood of the model. Various potential threshold values were tested and the ones that provided the best model fit indicated by the likelihood ratio test were chosen.

To explore the correlation between ln-SIRI and the SIRI quartile and length of stay in the hospital and the length of stay in the ICU, multivariate Linear regression analyses were conducted. To avoid the influence of different clinical outcomes at discharge, the analyses were conducted separately on all patients, patients who experienced in-hospital death, and patients who did not experience in-hospital death.

Then, the time-dependent receiver operating characteristic (TDROC) curves were used to examine the association of the SIRI quartile with short-term mortality. The areas under the TDROC curves were used to compare the predictive value of the risk stratification based on SIRI and the predictive value of the risk stratification based on the optimal cutoffs of NLR. The value of the optimal cutoffs of NLR is from the previous literature (10).

All statistical analyses were carried out using the software packages R (https://www.R-project.org, The R Foundation) and Free Statistical software. At *p* < 0.05, statistical differences were deemed significant.

## 3. Results

### 3.1. Baseline characteristics

The baseline characteristics of the patients are presented in Table 1. The mean age of the patients was 69.4 ± 13.2 years, with males accounting for approximately 62.7% of the sample. Within 30 days after admission, 677 patients (15.8%) had died, and within 90 days, 882 patients (20.6%) had died. In patients with elevated levels of SIRI, it is often observed that they are older males. The prevalence of comorbidities such as congestive heart failure, peripheral vascular disease, chronic obstructive pulmonary disease, rheumatic hypercholesterolemia, and hypertension is higher in this population. Additionally, there is a higher incidence of abnormal laboratory values. Furthermore, mortality rates are also elevated in these patients.

**Table 1:** The clinical characteristics of critically ill patients with AMI.

Characteristics	SIRI					*p*-value
	Total (*n* = 4,291)	Quartile 1 (*n* = 1,073)	Quartile 2 (*n* = 1,072)	Quartile 3 (*n* = 1,073)	Quartile 4 (*n* = 1,073)	
Demographics						
Age(year)	69.4 + 13.2	67.6 + 12.7	69.9 + 13.1	69.4 + 13.6	70.8 + 13.3	<0.001
Male gender, *n* (%)	2,689 (62.7)	703 (65.5)	680 (63.4)	654 (61)	652 (60.8)	0.072
Ethnicity, *n* (%)						<0.001
White	2,795 (65.1)	638 (59.5)	672 (62.7)	715 (66.6)	770 (71.8)	
Black	407 (9.5)	107 (10)	93 (8.7)	137 (12.8)	70 (6.5)	
Other	1,089 (25.4)	328 (30.6)	307 (28.6)	221 (20.6)	233 (21.7)	
Vital signs						
SBP (mmHg)	116.2 + 15.7	115.9 + 15.0	114.4 + 14.8	118.4 + 16.4	116.2 + 16.1	<0.001
DBP (mmHg)	61.8 + 11.1	61.6 + 10.8	62.8 + 10.9	62.1 + 10.9	60.8 + 11.5	<0.001
MBP (mmHg)	77.2 + 10.6	77.8 + 10.6	77.8 + 10.4	77.3 + 10.4	76.1 + 10.8	<0.001
HR (beats/min)	82.7 + 14.9	81.4 + 13.2	85.2 + 16.2	79.7 + 14.1	84.4 + 15.3	<0.001
RR (beats/min)	19.6 + 3.6	19.0 + 3.4	20.4 + 3.8	18.7 + 3.3	20.1 + 3.8	<0.001
Temperature (◦C)	36.7 + 0.6	36.7 + 0.5	36.8 + 0.5	36.7 + 0.5	36.7 + 0.7	<0.001
SpO2 (%)	96.8 + 2.6	97.0 + 2.7	96.5 + 2.8	97.0 + 2.1	96.7 + 2.6	<0.001
Comorbidities, *n* (%)						
CHF	2,079 (48.5)	448 (41.8)	593 (55.3)	468 (43.6)	570 (53.1)	<0.001
PVD	674 (15.7)	142 (13.2)	166 (15.5)	180 (16.8)	186 (17.3)	0.045
CVD	559 (13.0)	144 (13.4)	142 (13.2)	131 (12.2)	142 (13.2)	0.833
COPD	1,132 (26.4)	204 (19)	295 (27.5)	291 (27.1)	342 (31.9)	<0.001
Rheumatic disease	173 (4.0)	34 (3.2)	34 (3.2)	47 (4.4)	58 (5.4)	0.021
Diabetes	1,837 (42.8)	500 (46.6)	437 (40.8)	443 (41.3)	457 (42.6)	0.027
Paraplegia	118 (2.7)	31 (2.9)	38 (3.5)	21 (2)	28 (2.6)	0.157
Renal disease	1,349 (31.4)	299 (27.9)	395 (36.8)	322 (30)	333 (31)	<0.001
Malignant cancer	359 (8.4)	95 (8.9)	103 (9.6)	72 (6.7)	89 (8.3)	0.096
SLD	95 (2.2)	18 (1.7)	29 (2.7)	24 (2.2)	24 (2.2)	0.453
HC	266 (6.2)	43 (4)	38 (3.5)	93 (8.7)	92 (8.6)	<0.001
VT	289 (6.7)	58 (5.4)	99 (9.2)	49 (4.6)	83 (7.7)	<0.001
HTN	1,815 (42.3)	442 (41.2)	316 (29.5)	517 (48.2)	540 (50.3)	<0.001
Laboratory tests						
Hemoglobin (g/dl)	10.9 + 2.1	10.5 + 2.0	10.6 + 2.2	11.2 + 2.1	11.2 + 2.1	<0.001
WBC (10^9^/L)	11.9 (8.9,15.8)	11.0 (8.2,14.0)	14.0 (10.818.0)	9.2 (7.3,12.3)	13.8 (11.0,17.6)	<0.001
Platelet (10^9^/L)	208.1 + 96.2	177.8 + 75.7	203.0 + 95.2	214.7 + 92.3	236.7 + 109.1	<0.001
Glucose (mg/dl)	135.7 (118.0,170.9)	131.0 (119.0,154.8)	140.4 (120.0,185.1)	131.0 (114.3,161.0)	144.9 (120.0,185.0)	<0.001
Creatinine (mg/dl)	1.1 (0.8,1.9)	1.0 (0.8,1.5)	1.3 (0.9,2.1)	1.1 (0.8,1.6)	1.2 (0.9,2.0)	<0.001
Calcium (mmol/L)	8.4 + 0.8	8.5 + 0.8	8.4 + 0.8	8.5 + 0.8	8.4 + 0.9	<0.001
Chloride (mmol/L)	103.5 + 5.8	103.8 + 5.5	102.2 + 6.4	104.4 + 5.3	103.6 + 5.5	<0.001
Sodium (mmol/L)	138.1 + 4.5	138.3 + 4.5	138.0 + 5.0	138.3 + 4.1	137.9 + 4.4	0.164
PTT (sec)	33.8 (28.4,51.5)	32.7 (28.4,48.5)	33.5 (28.1,53.5)	33.3 (28.4,50.6)	36.2 (29.0,55.5)	0.001
Drug use, *n* (%)						
Warfarin	469 (10.9)	96 (8.9)	107 (10)	137 (12.8)	129 (12)	0.016
Aspirin	2,899 (67.6)	875 (81.5)	760 (70.9)	624 (58.2)	640 (59.6)	<0.001
BB	544 (12.7)	117 (10.9)	123 (11.5)	153 (14.3)	151 (14.1)	0.033
Outcomes, *n* (%)						
30-day mortality	677 (15.8)	99 (9.2)	257 (24)	122 (11.4)	199 (18.5)	<0.001
90-day mortality	882 (20.6)	133 (12.4)	328 (30.6)	156 (14.5)	265 (24.7)	<0.001
30-day re-admission	755 (17.6)	177 (16.5)	183 (17.1)	195 (18.2)	200 (18.6)	0.541
90-day re-admission	1,148 (26.8)	261 (24.3)	266 (24.8)	312 (29.1)	309 (28.8)	0.015
Los in hospital (days)	7.5 (4.3, 11.9)	8.0 (5.2, 11.8)	8.6 (4.8, 13.9)	5.8 (3.2, 9.7)	7.7 (4.5, 12.3)	< 0.001
Los in ICU (days)	2.1 (1.2, 4.0)	1.9 (1.2, 3.2)	2.7 (1.4, 4.9)	1.8 (1.1, 3.1)	2.7 (1.4, 5.0)	< 0.001

AMI, acute myocardial infarction; SIRI, systemic inflammation response index; SBP, systolic blood pressure; DBP, diastolic blood pressure; MBP, mean blood pressure; HR, heart rate; RR, respiratory rate; SpO2, percutaneous oxygen saturation; CHF, Congestive heart failure; PVD, Peripheral vascular disease; CVD, Cerebrovascular disease; COPD, chronic obstructive pulmonary disease; SLD, Severe liver disease; HC, Hypercholesterolemia; VT, Ventricular tachycardia; HTN, Hypertension; WBC, white blood cell; PTT, partial thromboplastin time; BB, Beta-blockers; ICU, Intensive Care Unit.

### 3.2. Relationship between the SIRI and the clinical outcomes

Univariate analysis revealed that age, gender, ethnicity, vital signs, drug utilization history, comorbidities, and laboratory parameters, including hemoglobin and glucose levels, were significantly associated with the 90-day mortality of patients diagnosed with AMI (Table 2).

**Table 2:** Univariable Cox regression analyses for outcomes in critically ill patients with AMI.

Variable	30-day mortality		90-day mortality	
	HR (95%CI)	*p*-value	HR (95%CI)	*p*-value
Demographics				
Age (year)	1.04 (1.03,1.04)	<0.001	1.04 (1.03,1.05)	<0.001
Male gender	0.7 (0.61,0.82)	<0.001	0.72 (0.63,0.83)	<0.001
Ethnicity (ref. = White)				
Black	0.75 (0.56,1.01)	0.062	0.67 (0.52,0.88)	0.004
Other	1.22 (1.03,1.44)	0.022	1.15 (0.99,1.34)	0.059
Vital signs				
SBP (mmHg)	0.97 (0.96,0.97)	<0.001	0.97 (0.96,0.97)	<0.001
DBP (mmHg)	0.98 (0.97,0.99)	<0.001	0.98 (0.97,0.99)	<0.001
MBP (mmHg)	0.96 (0.95,0.97)	<0.001	0.96 (0.96,0.97)	<0.001
HR (beats/min)	1.02 (1.02,1.03)	<0.001	1.02 (1.01,1.02)	<0.001
RR (beats/min)	1.17 (1.15,1.19)	<0.001	1.15 (1.13,1.17)	<0.001
Temperature (°C)	0.58 (0.53,0.65)	<0.001	0.63 (0.57,0.7)	<0.001
SpO2 (%)	0.88 (0.87,0.9)	<0.001	0.89 (0.88,0.9)	<0.001
Comorbidities,				
Congestive heart failure	1.52 (1.31,1.77)	<0.001	1.59 (1.39,1.82)	<0.001
Peripheral vascular disease	1.3 (1.07,1.58)	0.007	1.39 (1.18,1.64)	<0.001
Cerebrovascular disease	1.7 (1.41,2.06)	<0.001	1.69 (1.43,2)	<0.001
Chronic pulmonary disease	1.24 (1.06,1.46)	0.009	1.29 (1.11,1.48)	<0.001
Rheumatic disease	1.0,083 (0.6861,1.4817)	0.966	1.0071 (0.7182,1.4122)	0.967
Diabetes	0.92 (0.79,1.08)	0.303	0.94 (0.82,1.07)	0.357
Paraplegia	1.75 (1.22,2.51)	0.002	1.7 (1.23,2.35)	0.001
Renal disease	1.36 (1.16,1.58)	<0.001	1.41 (1.23,1.62)	<0.001
Malignant cancer	2.06 (1.67,2.55)	<0.001	2.42 (2.02,2.89)	<0.001
Severe liver disease	2.16 (1.49,3.14)	<0.001	1.94 (1.37,2.75)	<0.001
Hypercholesterolemia	0.89 (0.64,1.24)	0.497	0.82 (0.61,1.1)	0.19
Ventricular tachycardia	2.04 (1.62,2.58)	<0.001	2.04 (1.66,2.51)	<0.001
Hypertension	0.6 (0.51,0.71)	<0.001	0.6 (0.52,0.69)	<0.001
Laboratory tests				
Hemoglobin (g/dl)	0.91 (0.88,0.95)	<0.001	0.89 (0.86,0.92)	<0.001
Platelet (10^9^/L)	1.0001 (0.9993,1.0008)	0.887	1.0002 (0.9995,1.0008)	0.642
WBC (10^9^/L)	1.0099 (1.007,1.0129)	<0.001	1.0092 (1.0064,1.0121)	<0.001
Glucose (mg/dl)	1 (1,1.0001)	0.128	1 (1,1.0001)	0.13
Calcium (mmol/L)	0.68 (0.61,0.75)	<0.001	0.71 (0.65,0.78)	<0.001
Chloride (mmol/L)	0.98 (0.97,0.99)	0.002	0.98 (0.97,0.99)	0.001
Creatinine (mg/dl)	1.09 (1.06,1.11)	<0.001	1.08 (1.05,1.1)	<0.001
Sodium (mmol/L)	1.03 (1.02,1.05)	<0.001	1.03 (1.01,1.05)	<0.001
PTT (sec)	1.01 (1.01,1.01)	<0.001	1.01 (1.01,1.02)	<0.001
Drug use				
Warfarin	0.33 (0.22,0.48)	<0.001	0.44 (0.33,0.58)	<0.001
Aspirin	0.65 (0.56,0.76)	<0.001	0.67 (0.59,0.77)	<0.001
Beta-blockers	0.65 (0.5,0.84)	0.001	0.7 (0.56,0.87)	0.002

AMI, acute myocardial infarction; SBP, systolic blood pressure; DBP, diastolic blood pressure; MBP, mean blood pressure; HR, heart rate; RR, respiratory rate; SpO2, percutaneous oxygen saturation; WBC, white blood cell; Chronic pulmonary disease, chronic obstructive pulmonary disease; PTT, partial thromboplastin time.

**Figure 2:**
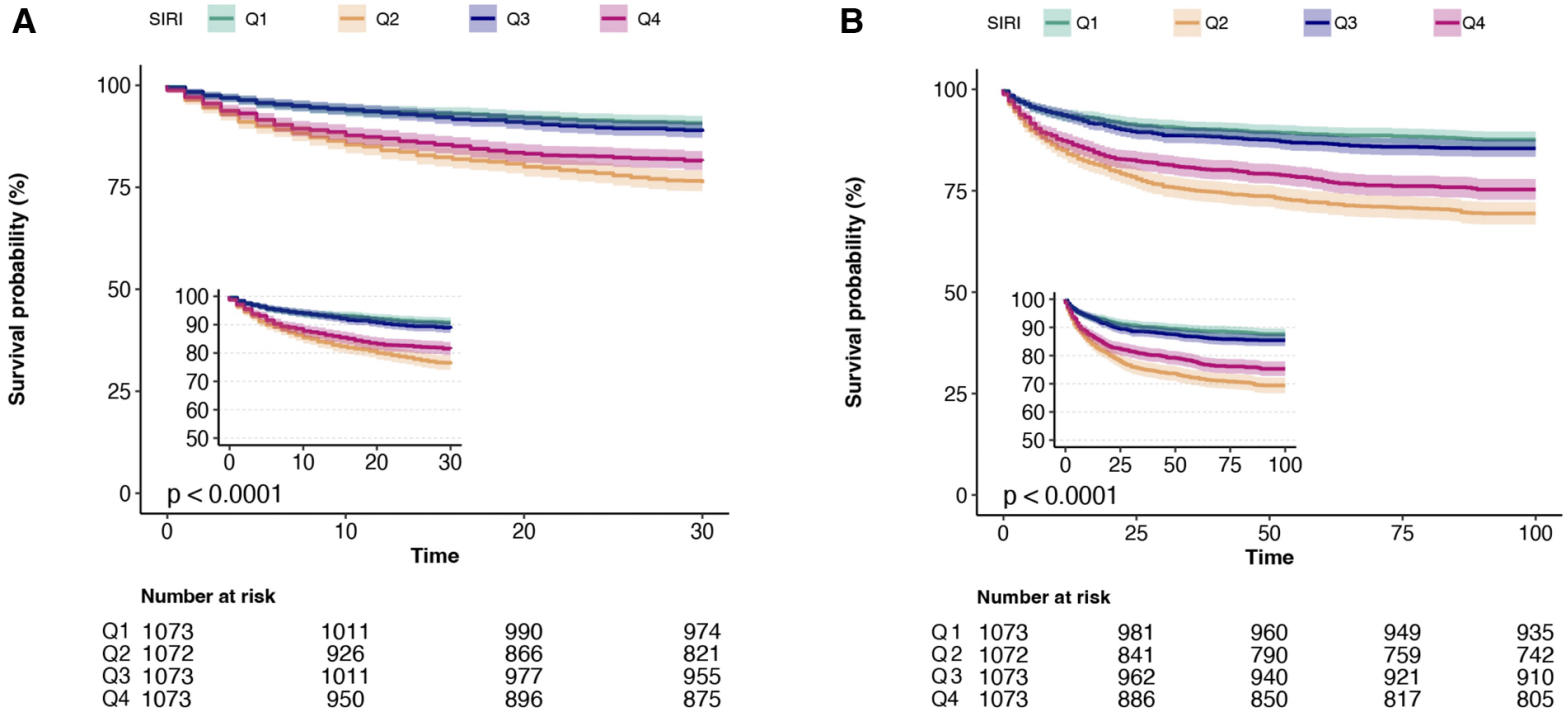
Kaplan–Meier survival curves of four groups based on the SIRI quartile for 30-day mortality and 90-day mortality. (**A**) 30-day mortality; (**B**) 90-day mortality.

In the multiple regression analysis (Table 3), in the non-adjusted model, ln-SIRI was associated with an increased risk of 30-day (HR = 1.050, 95% CI: 1.018–1.084, *p* = 0.0023) and 90-day (HR = 1.051, 95% CI: 1.022–1.08, *p* = 0.0004) all-cause mortality. In the multivariate model 1, after adjusting for age, gender, and ethnicity, ln-SIRI was also associated with the risk of 30-day (HR = 1.039, 95% CI: 1.006–1.073, *p* = 0.0191) and 90-day (HR = 1.038, 95% CI: 1.009–1.067, *p* = 0.0096) all-cause mortality. In model 2, after adjusting for the variables in model 2, ln-SIRI was associated with an increased risk of 30-day (HR = 1.035, 95% CI: 1.001–1.07, *p* = 0.0463) and 90-day (HR = 1.042, 95% CI: 1.012–1.072, *p* = 0.0064) all-cause mortality. However, a different trend in outcomes was found using the quartile of SIRI. The reference group for the SIRI quartiles is Q1. In all three models, Q2 and Q4 were significantly associated with an increased risk of 30-day and 90-day all-cause mortality, and there was no significant difference in risk between Q3 and Q1. The results of the Kaplan–Meier survival curves were consistent with the results of the multivariate regression analysis and showed that patients in Q2 had the highest mortality rate and those in Q1 had the lowest (Figure 2). Therefore, based on the results of the multivariate regression analysis and the Kaplan–Meier survival curves, we stratified the patients by risk of short-term mortality based on the quartiles of the SIRI as follows: Q1 was the low-risk group, Q3 was the medium-risk group, Q4 was the sub-high-risk group, and Q2 was the high-risk group.

**Table 3:** Multivariable Cox regression models of the association between SIRI and short-term mortality.

Variable	Unadjusted		Model 1		Model 2	
	crude.HR-95	crude. *p*-value	adj.HR-95CI	adj. *p*-value	adj.HR-95CI	adj. *p*-value
30-day mortality						
ln-SIRI	1.050 (1.018–1.084)	0.0023	1.039 (1.006–1.073)	0.0191	1.035 (1.001–1.07)	0.0463
SIRI quartile						
Q1	1(Ref)		1(Ref)		1(Ref)	
Q2	2.809 (2.227–3.542)	<0.001	2.64 (2.092–3.331)	<0.001	2.031 (1.604–2.571)	<0.001
Q3	1.238 (0.949–1.613)	0.115	1.191 (0.913–1.555)	0.198	1.225 (0.931–1.612)	0.1473
Q4	2.124 (1.669–2.703)	<0.001	1.951 (1.53–2.488)	<0.001	1.703 (1.32–2.195)	<0.001
90-day mortality						
ln-SIRI	1.051 (1.022–1.08)	0.0004	1.038 (1.009–1.067)	0.0096	1.042 (1.012–1.072)	0.0064
SIRI quartile						
Q1	1(Ref)		1(Ref)		1(Ref)	
Q2	2.748 (2.247–3.362)	<0.001	2.572 (2.102–3.148)	<0.001	2.063 (1.68–2.532)	<0.001
Q3	1.184 (0.939–1.492)	0.153	1.133 (0.898–1.43)	0.2921	1.212 (0.954–1.539)	0.1149
Q4	2.143 (1.74–2.64)	<0.001	1.945 (1.576–2.399)	<0.001	1.788 (1.435–2.227)	<0.001

Crude model: adjusted for none.

Model 1: adjusted for age, sex, and ethnicity.

Model 2: adjusted for age, sex, ethnicity, heart rate, systolic blood pressure, mean blood pressure, respiratory rate, percutaneous oxygen saturation, diabetes, hemoglobin, calcium, sodium, partial thromboplastin time, congestive heart failure, chronic obstructive pulmonary disease, malignant cancer, hypercholesterolemia, hypertension, aspirin, warfarin, beta-blockers.

**Figure 3:**
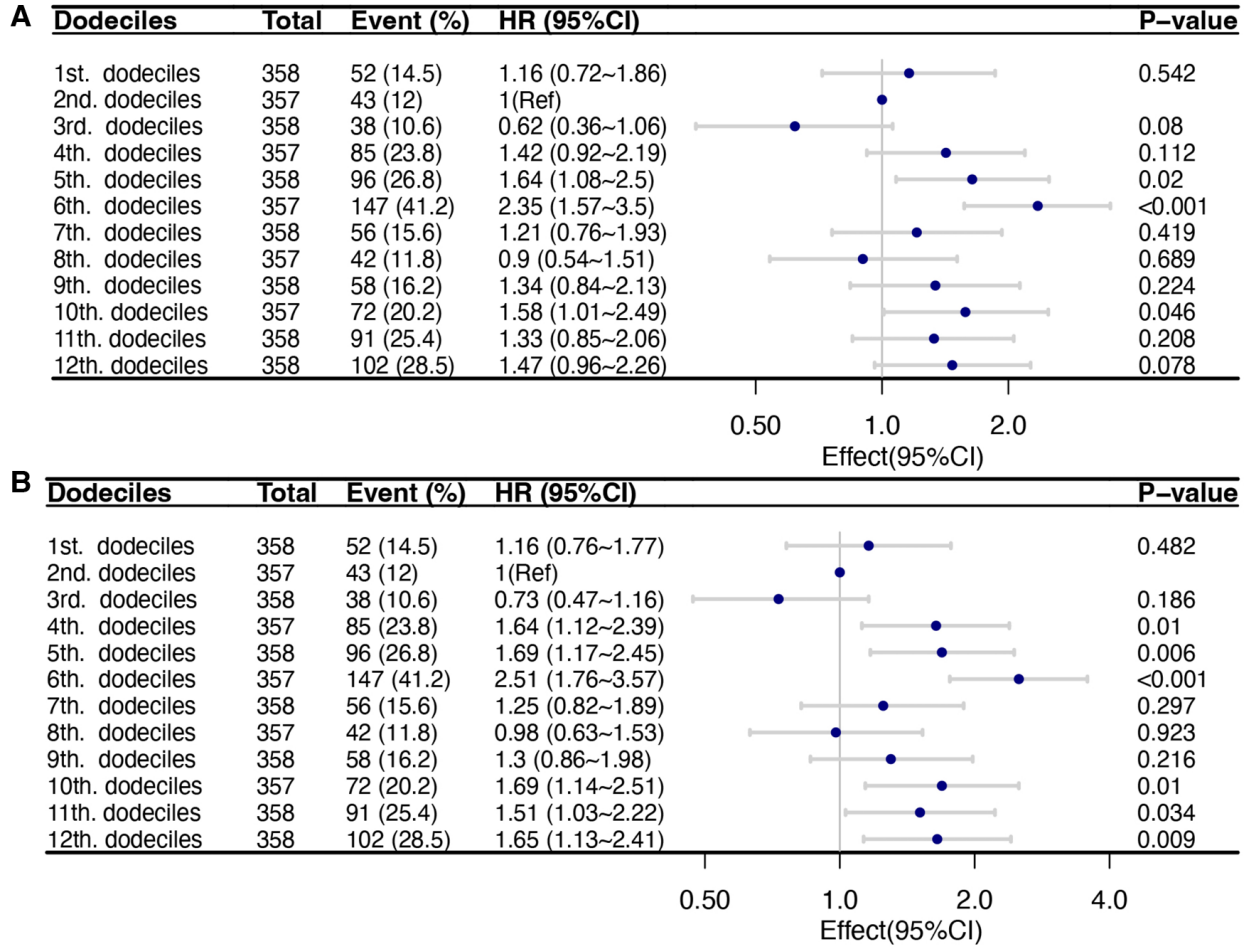
Multivariable Cox regression models of the association between the dodeciles of SIRI and short-term mortality of critical patients with AMI. The adjusted variables were consistent with Model 2 in the multivariate regression analysis. Dots indicate odds ratios (ORs), with horizontal lines indicating 95% CIs. (**A**) 30-day mortality; (**B**) 90-day mortality.

**Figure 4:**
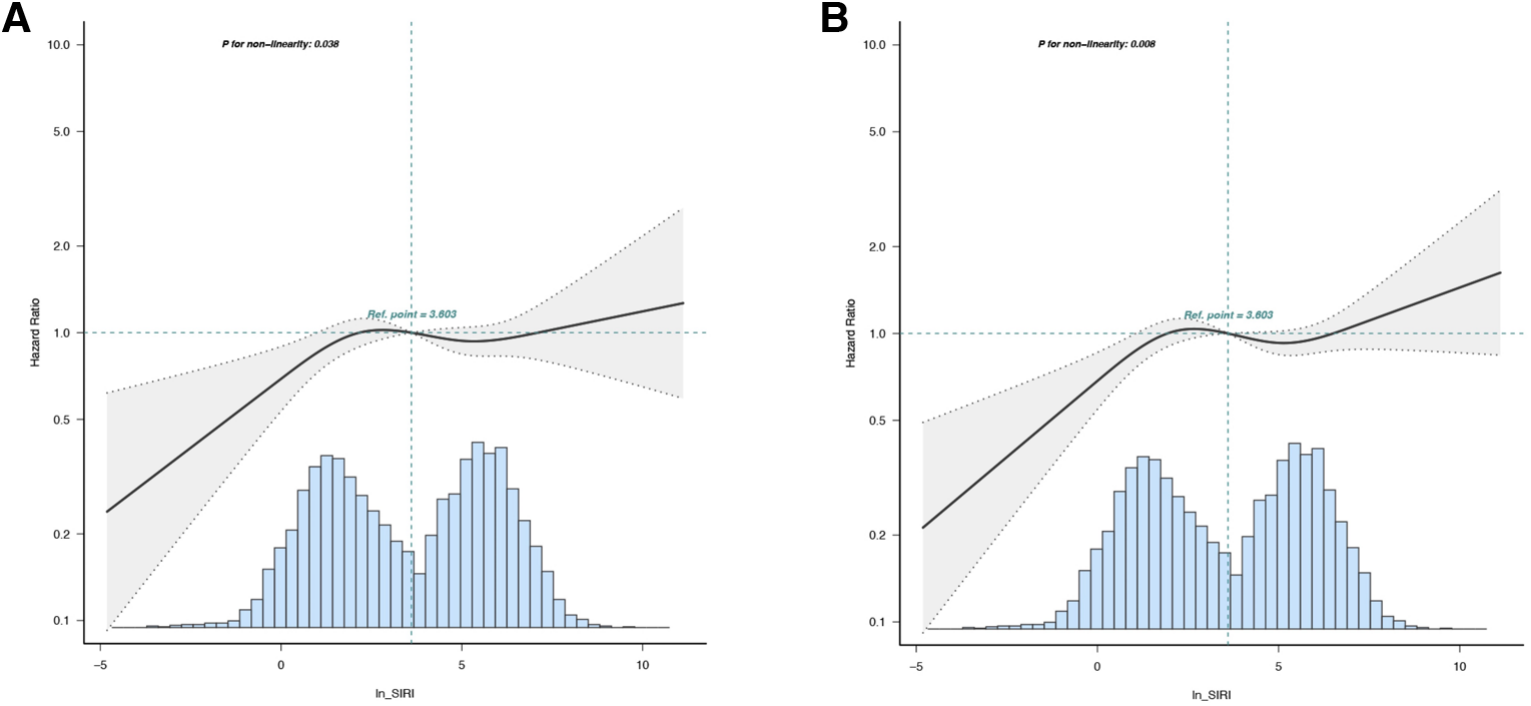
Nonlinear dose-response relationship between ln SIRI and short-term mortality. The adjusted variables were consistent with Model 2 in the multivariate regression analysis. The solid line and dashed line represent the estimated values and their corresponding 95% confidence intervals. (**A**) 30-day mortality; (**B**) 90-day mortality.

Then, to verify the reliability of the risk stratification, several analyses were conducted. Firstly, the SIRI was divided into 12 groups based on dodeciles, and a Cox multivariate regression analysis was performed. Q1 corresponds to 1st–3rd doceciles, Q2 corresponds to 4th–6th dodeciles, Q3 corresponds to 7th–9th dodeciles, and Q4 corresponds to 10th–12th dodeciles. The results are consistent with the risk stratification based on quartiles (Figure 3). Then, the restricted cubic spline analysis revealed a non-linear correlation between ln-SIRI and 30-day and 90-day all-cause mortality among AMI patients (*p*-values for non-linearity were 0.038 and 0.008, respectively) (Figure 4). The covariates that were adjusted for were consistent with Model 2 in the multivariate regression analysis. The threshold saturation effect demonstrated that, for 30-day mortality, when the ln-SIRI level is less than 2.9, the patient's mortality rate increases by 24.3% [odds ratio (OR): 1.243; 95% CI: 1.102–1.402; *p* < 0.001] for each unit increase in ln-SIRI. When the ln-SIRI is greater than 2.9 and less than 4.6, the patient's mortality rate decreases by 40.2% (OR: 0.598; 95% CI: 0.393–0.883; *p* = 0.0103) for each unit increases in ln-SIRI. When ln-SIRI > 4.6, the patient's mortality rate increases by 20.6% (OR: 1.206; 95% CI: 1.053–1.382; *p* = 0.0069) for each unit increase in ln-SIRI. For 90-day mortality, when the ln-SIRI level is less than 2.9, the patient's mortality rate increases by 24.2% (OR: 1.242; 95% CI: 1.12–1.378; *p* < 0.001) for each unit increase in ln-SIRI. When the ln-SIRI is greater than 2.9 and less than 4.6, the patient's mortality rate decreases by 47.3% (OR: 0.527; 95% CI: 0.37,0.751; *p* < 0.001) for each unit increases in ln-SIRI. When ln-SIRI > 4.6, the patient's mortality rate increases by 24.1% (OR: 1.241; 95% CI: 1.102–1.396; *p* < 0.001) for each unit increase in ln-SIRI (Table 4). Moreover, the distribution of the quartile groupings is consistent with thresholds. The first quartile, median, and third quartile are 1.5, 3.6, and 5.7, respectively. Therefore, Q1 is distributed to the left of the first threshold, Q2 and Q3 are near the two thresholds, and Q4 is to the right of the second threshold.

**Table 4:** Threshold effect analysis of the relationship of ln-SIRI (per change 1 unit) on mortality of critical AMI patients.

Threshold of ln SIRI	Adjusted OR (95% CI)	*p*-value
30-day mortality		
ln-SIRI < 2.9	1.243 (1.102,1.402)	<0.001
ln-SIRI > 2.9, < 4.6	0.589 (0.393,0.883)	0.0103
ln-SIRI > 4.6	1.206 (1.053,1.382)	0.0069
Likelihood Ratio test	–	<0.001
90-day mortality		
ln-SIRI < 2.9	1.242 (1.12,1.378)	<0.001
ln-SIRI > 2.9, < 4.6	0.527 (0.37,0.751)	<0.001
ln-SIRI > 4.6	1.241 (1.102,1.396)	<0.001
Likelihood Ratio test	–	<0.001

The adjusted variables were consistent with Model 2 in the multivariate Cox regression analysis.

For the secondary outcomes of the study, multiple regression analysis showed different results. In the Cox regression models (Supplementary Table S1), the results showed that there was no significant statistical relationship between 30-day readmission and 90-day readmission and ln-SIRI and SIRI quartiles. In the Linear regression models (Table 5), regarding the length of stay in the hospital, a significant negative association was found between ln-SIRI and hospital stay in the unadjusted model (coefficient: −0.178, 95% CI: −0.289 to −0.067, *p*-value: 0.0016), Model 1 (coefficient: −0.182, 95% CI: −0.293 to −0.070, *p*-value: <0.0014), and Model 2 (coefficient: −0.174, 95% CI: −0.298 to −0.049, *p*-value: 0.0063). When analyzing the SIRI quartiles, Q3 showed a significant negative association in the unadjusted model (coefficient: −2.178, 95% CI: −2.928 to −1.428, *p*-value: <0.001), Model 1 (coefficient: −2.216, 95% CI: −2.97 to −1.462, *p*-value: <0.001), and Model 2 (coefficient: −1.999, 95% CI: −2.834 to −1.165, *p*-value: <0.001). This means that the length of stay in the hospital, which decreased with increasing ln-SIRI, decreased significantly in patients in Q3 compared to patients in Q1. Although this trend was not significant among patients in Q2 and Q4. Regarding the length of stay in the ICU, a significant positive association was found between ln-SIRI and ICU stay in the unadjusted model (coefficient: 0.119, 95% CI: 0.059 to 0.18, *p*-value: 0.0001), Model 1 (coefficient: 0.127, 95% CI: 0.066 to 0.188, *p*-value: < 0.001), and Model 2 (coefficient: 0.078, 95% CI: 0.009 to 0.146, *p*-value: 0.0277). In terms of SIRI quartiles, significant positive associations were observed for Q2 and Q4 in all models, while no significant associations were found for Q3. The relationship was consistent with the results of risk stratification. Patients in Q2 and Q4 had longer lengths of stay in ICU, whereas patients in Q1 and Q3 had shorter lengths of stay in ICU. These results are consistent with the results of the analysis of patients who did not experience in-hospital death (Table 6). However, in patients who experienced in-hospital death, the results showed that there was no significant statistical relationship between the length of stay in the hospital and ICU and ln-SIRI and SIRI quartiles (Supplementary Table S2).

**Table 5:** Multivariable linear regression models of the association between SIRI and length of stay in hospital and length of stay in ICU in acute myocardial infarction patients.

Variable	Unadjusted		Model 1		Model 2	
	crude. Coefficient-95CI	crude. *p*-value	adj. Coefficient-95CI	adj. *p*-value	adj. Coefficient-95CI	adj. *p*-value
Length of stay in hospital						
ln-SIRI	−0.178 (−0.289–−0.067)	0.0016	−0.182 (−0.293–−0.07)	0.0014	−0.174 (−0.298–−0.049)	0.0063
SIRI quartile						
Q1	0(Ref)		0(Ref)		0(Ref)	
Q2	0.832 (0.082–1.583)	0.0298	0.84 (0.087–1.592)	0.0288	0.334 (−0.467–1.135)	0.4135
Q3	−2.178 (−2.928–−1.428)	<0.001	−2.216 (−2.97–−1.462)	<0.001	−1.999 (−2.834–−1.165)	<0.001
Q4	0.161 (−0.59–0.911)	0.6747	0.152 (−0.604–0.908)	0.6932	−0.126 (−0.961–0.709)	0.7669
Length of stay in ICU						
ln-SIRI	0.119 (0.059–0.18)	0.0001	0.127 (0.066–0.188)	<0.001	0.078 (0.009–0.146)	0.0277
SIRI quartile						
Q1	0(Ref)		0(Ref)		0(Ref)	
Q2	1.042 (0.632–1.452)	<0.001	1.080 (0.670–1.491)	<0.001	0.685 (0.243–1.128)	0.0024
Q3	−0.208 (−0.618–0.202)	0.3209	−0.165 (−0.576–0.247)	0.4327	−0.182 (−0.644–0.279)	0.4392
Q4	1.431 (1.021–1.841)	<0.001	1.489 (1.077–1.902)	<0.001	0.989 (0.528–1.451)	<0.001

The adjusted variables were consistent with Models in the multivariate Cox regression analysis.

**Table 6:** Multivariable linear regression models of the association between SIRI and length of stay in hospital and length of stay in ICU in acute myocardial infarction patients who did not experience in-hospital death.

Variable	Unadjusted		Model 1		Model 2	
	crude. Coefficient-95CI	crude. *p*-value	adj. Coefficient-95CI	adj. *p*-value	adj. Coefficient-95CI	adj. *p*-value
Length of stay in hospital						
ln-SIRI	−0.16 (−0.27–−0.05)	0.006	−0.16 (−0.27–−0.04)	0.007	−0.15 (−0.28–−0.02)	0.026
SIRI quartile						
Q1	0(Ref)		0(Ref)		0(Ref)	
Q2	1.32 (0.55–2.08)	0.001	1.32 (0.55–2.08)	0.001	0.69 (−0.13–1.51)	0.099
Q3	−2.42 (−3.18–−1.66)	<0.001	−2.44 (−3.21–−1.67)	<0.001	−2.18 (−3.04–−1.32)	<0.001
Q4	0.18 (−0.58–0.95)	0.639	0.2 (−0.57–0.97)	0.613	−0.2 (−1.06–0.66)	0.649
Length of stay in ICU						
ln-SIRI	0.11 (0.05–0.17)	<0.001	0.12 (0.06–0.18)	<0.001	0.07 (0.003–0.14)	0.0412
SIRI quartile						
Q1	0(Ref)		0(Ref)		0(Ref)	
Q2	0.99 (0.59–1.39)	<0.001	1.02 (0.62–1.42)	<0.001	0.59 (0.15–1.03)	0.009
Q3	−0.22 (−0.61–0.18)	0.29	−0.17 (−0.57–0.23)	0.41	−0.18 (−0.64–0.28)	0.442
Q4	1.25 (0.85–1.65)	<0.001	1.32 (0.92–1.72)	<0.001	0.81 (0.35–1.27)	0.001

The adjusted variables were consistent with Models in the multivariate Cox regression analysis.

### 3.3. TDROC curve analysis for short-term mortality

**Figure 5:**
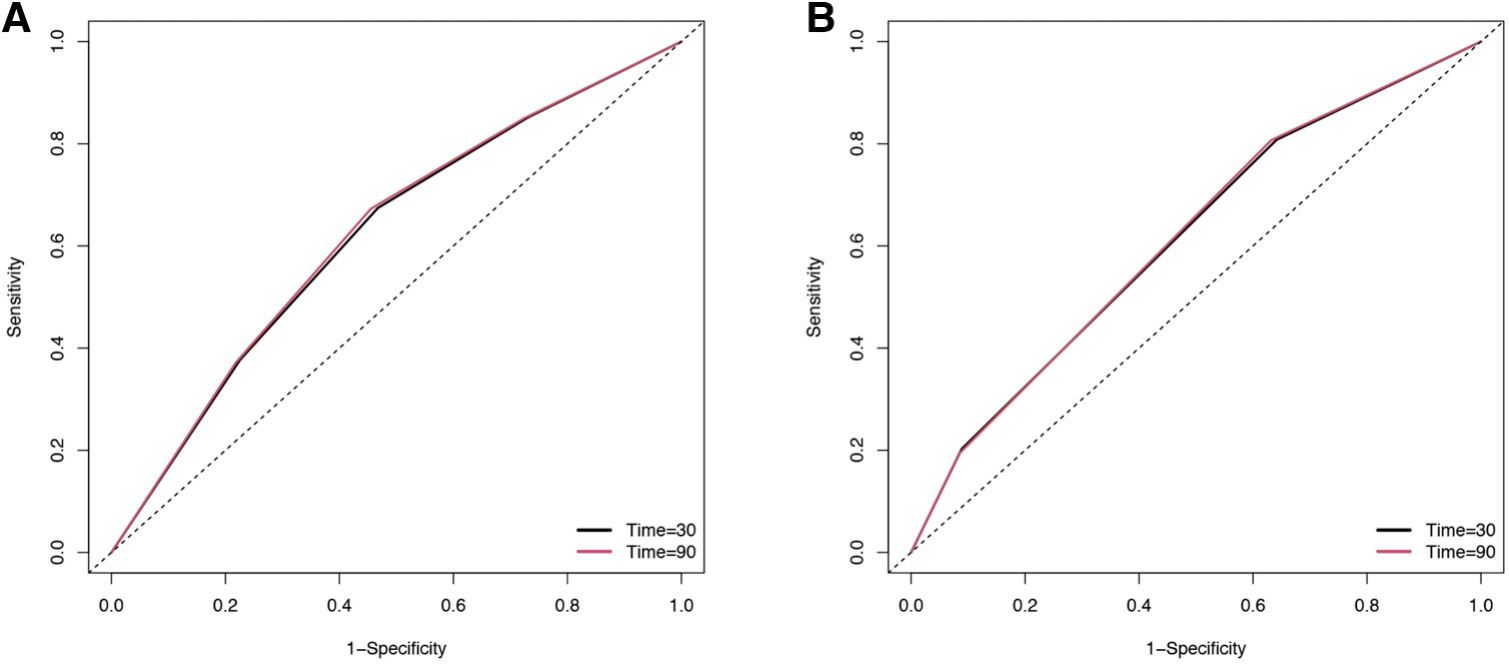
The time-dependent receiver operating characteristic (TDROC) curves of the predictive value of inflammatory indexes for short-term mortality in patients with AMI (**A**) risk stratification based on SIRI. (**B**) Risk stratification based on NLR.

TDROC curves were used to compare the predictive value of the risk stratification based on ln-SIRI and the risk stratification based on optimal cutoffs of NLR (10) (Figure 5 and Table 7). In the risk stratification based on ln-SIRI, for the 30-day mortality, the AUC is 0.6198, and the Max Youden is 0.2068. For 90-day mortality, the AUC is 0.6242, and the Max Youden is 0.2175. In the risk stratification based on NLR, for the 30-day mortality, the AUC is 0.6123, and the Max Youden is 0.1664. For 90-day mortality, the AUC is 0.6145, and the Max Youden is 0.175. Therefore, the risk stratification based on ln-SIRI appears to perform better than the risk stratification based on NLR in terms of overall accuracy and balance between sensitivity and specificity for both 30-day mortality and 90-day mortality.

**Table 7:** The time-dependent receiver operating curves (TDROC) for the predictive value of inflammatory indexes for short-term mortality in patients with AMI.

Stratification Method	Time (days)	AUC	Max Youden
SIRI	30	0.6198	0.2068
SIRI	90	0.6242	0.2175
NLR	30	0.6123	0.1664
NLR	90	0.6145	0.175

AUC, the area under the curve; SIRI, systemic inflammation response index; NLR, neutrophil-lymphocyte ratio.

### 3.4. Sensitivity analysis

The extent of missing data varied from 0 to 7.6% across the distinct variables. The distributions of any variable exhibiting missing data were the same between the imputation datasets and the observed complete case data (Supplementary Table S3).

Multiple regression analyses using only subjects with complete data gave similar results to those undertaken on the multiple imputed datasets (Supplementary Table S4).

To constrain the distribution range of the data and enhance its distributional pattern, thereby facilitating the interpretation of the curve-fitting outcomes, we conducted an analysis of the natural logarithm of SIRI (ln-SIRI) in our study. Even after restoring the results of threshold saturation effects to SIRI for analysis, the obtained results remained robust and consistent (Supplementary Table S5).

## 4. Discussion

This investigation delved into the correlation between SIRI and unfavorable prognosis in patients afflicted with AMI. Following meticulous adjustment for multiple variables via a Cox regression model and smooth curve fitting, it was ascertained that SIRI was significantly linked to all-cause mortality in AMI patients. Upon scrutinizing the threshold saturation effect, it was discovered that when the ln-SIRI level is below 2.9, each unit increases in ln-SIRI resulting in a 20.7% escalation in the patient's mortality rate. When ln-SIRI surpasses 2.9 but remains below 4.6, each unit increase in ln-SIRI leads to a 49.2% reduction in the patient's mortality rate. When ln-SIRI exceeds 4.6, each unit increases in ln-SIRI results in a 23.9% surge in the patient's mortality rate. For the SIRI quartile, patients in Q2 and Q4 had significantly higher mortality than those in Q1 and Q3. There was no significant statistical relationship between 30-day readmission and 90-day readmission and SIRI. However, patients in Q3 had shorter lengths of stay in the hospital. Patients had longer lengths of stay in the ICU stays in Q2 and Q4. Furthermore, the risk stratification based on SIRI appears to perform better than the risk stratification based on NLR on predictive value for short-term mortality. We also performed analysis on only subjects with complete data and the results remained stable. We also restored ln-SIRI to SIRI and performed data analysis, and the results also showed stability.

AMI is a formidable global health challenge, accounting for nearly 1.8 million deaths annually and representing a staggering 20% of all mortalities in Europe (10). Given its high incidence and dire prognosis, AMI poses a grave threat to public health. The condition can trigger a cascade of serological changes and incite an inflammatory response, culminating in deleterious cardiovascular events and ultimately, patient demise (11, 12). As such, it is imperative for clinical practitioners to identify serological markers capable of detecting high-risk AMI patients in a timely manner.

Numerous investigations have underscored the significance of inflammation in the genesis of atherosclerotic plaques. Firstly, inflammation exerts a profound influence on all stages of atherosclerotic plaque development and can precipitate thrombotic events (13). Secondly, monocytes adhere to the vascular endothelium and transmigrate into the arterial intima where they differentiate into foamy macrophages—a pivotal event in the nascent initiation of plaque formation (14, 15). Moreover, monocytes play a crucial role in neovascularization within the arterial wall and atherosclerotic plaques, a process that assumes a critical role in the pathogenesis of myocardial infarctions and strokes (16). Additionally, neutrophils infiltrate coronary plaques and myocardial tissue during AMI (17).

In numerous diseases, SIRI has emerged as an independent prognostic indicator, including acute ischemic stroke, heart failure, traumatic brain injury, breast cancer, and acute pancreatitis (18–22). It's a composite index covering a broader scope than the two composite inflammatory indices, namely the monocyte-to-lymphocyte ratio (MLR) and the neutrophil-to-lymphocyte ratio (NLR). Previous investigations have demonstrated that MLR and NLR are linked to a variety of ailments. For instance, NLR and MLR have been linked to afflictions such as sepsis and community-acquired pneumonia (23). In the case of coronary artery disease, antecedent research has also revealed that elevated admission NLR is an autonomous risk factor for an unfavorable prognosis in patients with acute myocardial infarction (AMI) (24); patients with low admission MLR exhibit higher one-year mortality rates (25). As a composite index that encompasses both NLR and MLR, SIRI is capable of more comprehensively and accurately reflecting the inflammatory response of patients.

Furthermore, past studies have also demonstrated that the value of MLR and NLR is not limited to predicting patient prognosis, but also has value in guiding drug therapy. For example, Nebivolol can reduce NLR (26), and Amlodipine and Valsartan can lower NLR in hypertensive patients (27). Therefore, SIRI may not only serve as an indicator for predicting patient outcomes but may also hold great potential in guiding treatment.

The primary advantage of our investigation lies in its reliance on extensive, authentic data, thereby augmenting the dependability of our discoveries. Nevertheless, our study was not without limitations. Firstly, we excluded patients for whom SIRI values could not be calculated due to missing data, which may have resulted in selection bias. Secondly, we excluded patients without the first admission. Because recurrent AMI has a low incidence in 1 year and has different clinical characteristics from patients with initial AMI (28). This could potentially confound the association between SIRI and short-term mortality in our study. However, this exclusion criterion may limit the generalizability of our findings to all AMI patients. Future studies should investigate the association between SIRI and short-term mortality in all AMI patients. Thirdly, our study was a retrospective, observational study conducted at a single center with possible selection bias. The relationship between SIRI and the mortality of patients with AMI needs to be further validated in the future by prospective cohort studies, and external generalizability needs to be investigated in diverse populations and countries. Furthermore, our research omitted super-acute complications such as stunned heart syndrome, pneumonia, or sepsis, as the ICD code represents a definitive diagnosis. Consequently, the SIRS index may have been inflated. Lastly, it is crucial to compare the risk stratification based on SIRI with other composite inflammatory indices to verify which index has superior predictive value. However, due to the lack of relevant reports, only risk stratification based on NLR was compared with SIRI. In the future, comparing the risk stratification based on SIRI with other composite inflammatory markers is important, such as MLR.

## 5. Conclusion

In conclusion, our study provides compelling evidence that SIRI is a significant predictor of all-cause mortality in patients with AMI. Our findings suggest that SIRI may serve as a valuable tool for risk stratification and prognostication in this patient population. The results of the threshold saturation effect provide further evidence of this relationship. Additionally, our analysis suggests that SIRI may outperform NLR in predicting short-term mortality risk. Our study also sheds light on the potential clinical implications of SIRI, as patients in certain SIRI quartiles were found to have shorter or longer hospital stays. Overall, our findings contribute to a better understanding of the role of SIRI in AMI prognosis and may inform the development of more effective risk stratification strategies for this patient population.

## Data Availability

The original contributions presented in the study are included in the article/**Supplementary Material**, further inquiries can be directed to the corresponding author.
