# Supplementary material for: A nonlinear relationship between systemic inflammation response index and short-term mortality in patients with acute myocardial infarction: a retrospective study from MIMIC-IV
Source: Front Cardiovasc Med. 2023 Jul 24;10:1208171. doi: 10.3389/fcvm.2023.1208171 (PMC10406293; doi:10.3389/fcvm.2023.1208171)
Supplement: Supplementary file 1 [file Table1.docx]

Supplementary Table 1. Multivariable Cox regression models of the association between SIRI and short-term readmission.

| Variable |  | Unadjusted | |  | Model 1 | |  | Model 2 | |
| --- | --- | --- | --- | --- | --- | --- | --- | --- | --- |
|  |  | crude.HR-95CI | crude. P-value |  | adj.HR-95CI | adj. P-value |  | adj.HR-95CI | adj. P-value |
| 30-day re-admission |  |  |  |  |  |  |  |  |  |
| ln-SIRI |  | 0.982 (0.954~1.011) | 0.2257 |  | 0.982 (0.953~1.011) | 0.2157 |  | 0.992 (0.96~1.026) | 0.6491 |
| SIRI quartile |  |  |  |  |  |  |  |  |  |
| Q1 |  | 1(Ref) |  |  | 1(Ref) |  |  | 1(Ref) |  |
| Q2 |  | 1.05 (0.854~1.291) | 0.6449 |  | 1.121 (0.897~1.402) | 0.3146 |  | 1.121 (0.897~1.402) | 0.3146 |
| Q3 |  | 0.889 (0.726~1.09) | 0.2584 |  | 1.005 (0.798~1.266) | 0.9643 |  | 1.005 (0.798~1.266) | 0.9643 |
| Q4 |  | 0.923 (0.754~1.13) | 0.4395 |  | 0.966 (0.768~1.217) | 0.771 |  | 0.966 (0.768~1.217) | 0.771 |
| 90-day re-admission |  |  |  |  |  |  |  |  |  |
| ln-SIRI |  | 0.994 (0.971~1.018) | 0.6119 |  | 0.995 (0.971~1.019) | 0.6624 |  | 1.004 (0.977~1.032) | 0.7856 |
| SIRI quartile |  |  |  |  |  |  |  |  |  |
| Q1 |  | 1(Ref) |  |  | 1(Ref) |  |  | 1(Ref) |  |
| Q2 |  | 1.119 (0.942~1.328) | 0.2 |  | 1.122 (0.944~1.334) | 0.1899 |  | 1.198 (0.993~1.447) | 0.0598 |
| Q3 |  | 1.016 (0.861~1.198) | 0.8527 |  | 1.018 (0.862~1.202) | 0.8315 |  | 1.117 (0.923~1.352) | 0.2553 |
| Q4 |  | 0.98 (0.831~1.156) | 0.8139 |  | 0.99 (0.837~1.171) | 0.906 |  | 1.049 (0.867~1.268) | 0.6249 |

Crude model: adjusted for none.

Model 1: adjusted for age, sex, and ethnicity.

Model 2: adjusted for age, sex, ethnicity, heart rate， systolic blood pressure， mean blood pressure， respiratory rate， percutaneous oxygen saturation， diabetes， hemoglobin, calcium, sodium, partial thromboplastin time, congestive heart failure, chronic obstructive pulmonary disease, malignant cancer, hypercholesterolemia, hypertension, aspirin, warfarin, beta-blockers.

Supplementary Table 2. Multivariable Linear regression models of the association between SIRI and length of stay in hospital and length of stay in ICU in acute myocardial infarction patients who experienced in-hospital death.

| Variable |  | Unadjusted | |  | Model 1 | |  | Model 2 | |
| --- | --- | --- | --- | --- | --- | --- | --- | --- | --- |
|  |  | crude. Coefficient-95CI | crude. P-value |  | adj. Coefficient-95CI | adj. P-value |  | adj. Coefficient-95CI | adj. P-value |
| length of stay in hospital | |  |  |  |  |  |  |  |  |
| ln-SIRI | | -0.28 (-0.67~0.11) | 0.155 |  | -0.27 (-0.66~0.12) | 0.176 |  | -0.403 (-0.81~0.004) | 0.0527 |
| SIRI quartile | |  |  |  |  |  |  |  |  |
| Q1(-4.8～1.5） |  | 0(Ref) |  |  | 0(Ref) |  |  | 0(Ref) |  |
| Q2(1.5～3.6) |  | -0.25 (-2.95~2.46) | 0.859 |  | -0.28 (-2.99~2.42) | 0.838 |  | -0.54 (-3.19~2.11) | 0.69 |
| Q3(3.6~5.7) |  | -0.42 (-3.13~2.29) | 0.762 |  | -0.44 (-3.15~2.27) | 0.751 |  | -1 (-3.71~1.71) | 0.47 |
| Q4(5.7~11.1) |  | -0.63 (-3.33~2.08) | 0.649 |  | -0.55 (-3.26~2.16) | 0.693 |  | -1.2 (-4~1.59) | 0.399 |
| length of stay in ICU | |  | | | | | |  |  |
| ln-SIRI |  | 0.13 (-0.11~0.37) | 0.298 |  | 0.15 (-0.09~0.4) | 0.218 |  | -0.02 (-0.27~0.23) | 0.859 |
| SIRI quartile |  | | | | | | | | |
| Q1 |  | 0(Ref) |  |  | 0(Ref) |  |  | 0(Ref) |  |
| Q2 |  | 0.05 (-1.63~1.74) | 0.95 |  | 0 (-1.68~1.68) | 0.999 |  | -0.21 (-1.84~1.42) | 0.8 |
| Q3 |  | 0.03 (-1.65~1.72) | 0.969 |  | 0.21 (-1.47~1.89) | 0.804 |  | -0.68 (-2.35~0.99) | 0.425 |
| Q4 |  | 0.78 (-0.9~2.47) | 0.364 |  | 0.87 (-0.81~2.56) | 0.311 |  | 0.07 (-1.65~1.79) | 0.935 |

The adjusted variables were consistent with Models in the multivariate Cox regression analysis.

Supplementary Table 3. Distributions of variables with missing data comparing observed complete case data to results from pooling the datasets with imputed variables from multiple imputation

| Variable | Level/Unit | Number (%) with missing data | Complete case | Multiple imputation |
| --- | --- | --- | --- | --- |
| DBP (mmHg) | Mean+SD | 27（0.63） | 61.8 + 11.1 | 61.8 + 11.1 |
| MBP (mmHg) | Mean+SD | 11（0.26） | 77.2 + 10.6 | 77.2 + 10.6 |
| SBP (mmHg) | Mean+SD | 27（0.63） | 116.2 + 15.6 | 116.2 + 15.7 |
| Calcium (mmol/L) | Mean+SD | 326（7.60） | 8.4 + 0.8 | 8.4 + 0.8 |
| Chloride (mmol/L) | Mean+SD | 1（0.02） | 103.5 + 5.8 | 103.5 + 5.8 |
| HR (beats/min) | Mean+SD | 10（0.23） | 82.7 + 14.9 | 82.7 + 14.9 |
| Hemoglobin (g/dL) | Mean+SD | 1（0.02） | 10.9 + 2.1 | 10.9 + 2.1 |
| RR (beats/min) | Mean+SD | 13（0.30） | 19.6 + 3.6 | 19.6 + 3.6 |
| Sodium (mmol/L) | Mean+SD | 1（0.02） | 138.1 + 4.5 | 138.1 + 4.5 |
| SpO2 (%) | Mean+SD | 13（0.30） | 96.8 + 2.6 | 96.8 + 2.6 |
| Temperature (_C) | Mean+SD | 190（4.43） | 36.8 + 0.6 | 36.7 + 0.6 |
| Glucose (mg/dL) | Median（IQR） | 45（1.05） | 135.9 (118.1, 171.0) | 135.7 (118.0, 170.9) |

SBP, systolic blood pressure; DBP, diastolic blood pressure; MBP, mean blood pressure; HR, heart rate; RR, respiratory rate; SpO2, percutaneous oxygen saturation.

Supplementary Table 4. Multivariable Cox regression models evaluating the association between ln-SIRI and 90-day all-cause mortality with complete case data.

| Variable | Unadjusted | |  | Model 1 | |  | Model 2 | |
| --- | --- | --- | --- | --- | --- | --- | --- | --- |
|  | crude.HR_95CI | P-value |  | adj.HR_95CI | P-value |  | adj.HR_95CI | P-value |
| ln-SIRI | 1.051 (1.022~1.08) | 0.0004 |  | 1.038 (1.009~1.067) | 0.0094 |  | 1.04 (1.009~1.071) | 0.0099 |

Crude model: adjusted for none.

Model 1: adjusted for age, sex, and ethnicity.

Model 2: adjusted for age, sex, ethnicity, heart rate， systolic blood pressure， mean blood pressure， respiratory rate， percutaneous oxygen saturation， diabetes， hemoglobin, calcium, sodium, congestive heart failure, chronic obstructive pulmonary disease, malignant cancer, hypercholesterolemia, hypertension, aspirin, warfarin, beta-blockers.

Supplementary Table 5. Threshold effect analysis of the relationship of SIRI (per change 1 unit) on 90-day mortality of critical AMI patients.

| Threshold of ln-SIRI | Adjusted OR (95% CI) | P-value |
| --- | --- | --- |
| ln-SIRI<2.9 | 1.063 (1.041,1.086) | < 0.001 |
| ln-SIRI>2.9, <4.6 | 0.9907 (0.986,0.9954) | < 0.001 |
| ln-SIRI>4.6 | 1.0006 (1.0001,1.001) | 0.0083 |
| Likelihood Ratio test | - | <0.001 |

The adjusted variables were consistent with Model 2 in the multivariate regression analysis.
